# Eye Movement Abnormalities in Multiple Sclerosis: Pathogenesis, Modeling, and Treatment

**DOI:** 10.3389/fneur.2018.00031

**Published:** 2018-02-05

**Authors:** Alessandro Serra, Clara G. Chisari, Manuela Matta

**Affiliations:** ^1^Neurology, Louis Stokes VA Medical Center, University Hospitals and Case Western Reserve School of Medicine, Cleveland, OH, United States; ^2^Neurology, University of Catania, Catania, Italy; ^3^Neurology, Ospedale San Luigi Gonzaga, Orbassano, Italy

**Keywords:** eye movements, multiple sclerosis, internuclear ophthalmoplegia, nystagmus, pathologic saccades

## Abstract

Multiple sclerosis (MS) commonly causes eye movement abnormalities that may have a significant impact on patients’ disability. Inflammatory demyelinating lesions, especially occurring in the posterior fossa, result in a wide range of disorders, spanning from acquired pendular nystagmus (APN) to internuclear ophthalmoplegia (INO), among the most common. As the control of eye movements is well understood in terms of anatomical substrate and underlying physiological network, studying ocular motor abnormalities in MS provides a unique opportunity to gain insights into mechanisms of disease. Quantitative measurement and modeling of eye movement disorders, such as INO, may lead to a better understanding of common symptoms encountered in MS, such as Uhthoff’s phenomenon and fatigue. In turn, the pathophysiology of a range of eye movement abnormalities, such as APN, has been clarified based on correlation of experimental model with lesion localization by neuroimaging in MS. Eye movement disorders have the potential of being utilized as structural and functional biomarkers of early cognitive deficit, and possibly help in assessing disease status and progression, and to serve as platform and functional outcome to test novel therapeutic agents for MS. Knowledge of neuropharmacology applied to eye movement dysfunction has guided testing and use of a number of pharmacological agents to treat some eye movement disorders found in MS, such as APN and other forms of central nystagmus.

## Introduction

Multiple sclerosis (MS) is a common disorder of the central nervous system (CNS) that affects more than 2 million people worldwide. Once thought to be predominantly an autoimmune inflammatory disease, MS is now regarded as a complex entity characterized by inflammatory demyelinating events and a significant component of neurodegeneration that manifests as neuronal and axonal loss since the early stages of the disease. Our understanding of the disease has evolved dramatically over the years and, while MS was typically considered an immune disease targeting the white matter and due to T-cells dysfunction, it is now clear that the pathological process targets both the gray and white matter and is enacted by complex involvement and dynamics of multiple cells, including T and B-cells, macrophages, mast cells, etc. The typical variability of phenotype and disease course observed in MS, spanning from relapsing to primary or secondary progressive clinical scenarios, is probably due to different extent and combination of inflammatory and neurodegenerative processes involving various CNS areas.

Multiple sclerosis is the main cause of non-traumatic disability of young adults, and it has profound functional consequences on men and women who are often just beginning to start families and advance in their career. Disability in MS is quantified using standard scales such as the expanded disability status scale and the multiple sclerosis functional composite (1). However, such scales have limitations, especially when it comes to addressing disability arising from eye movement dysfunction, a common cause of transient or long-term impairment in MS. The presence of eye movement abnormalities correlate with greater level of disability in affected patients and generally predict a worse prognosis (2). Poor scores at automated tests of saccadic performance, such as the King-Devick (K-D) test of rapid number naming, also correlate with higher levels of disability (3). While some additional scales, such as the 25-Item National Eye Institute Visual Functioning Questionnaire and a 10-Item Neuro-Ophthalmic Supplement (4), can help track visual symptoms such as defects in binocular vision, blurred vision and diplopia, no standard evaluation includes a systematic approach for testing functional classes of eye movements in MS. Even without a formal standardized tool, an accurate bedside eye movement examination can aid or support the diagnosis of MS, for example, by providing evidence of disease dissemination in space (5). As the physiology and underlying anatomical network of eye movement control is well known from animal models and studies in humans (6), eye movement abnormalities are highly localizing to CNS structural lesions. Eye movement recording and analysis are required for more detailed quantitative characterization. One of the advantages of studying eye movements in the laboratory setting is that they can be precisely measured over time. Using such approach, for example, internuclear ophthalmoparesis has been proposed as a model for studying the effect of increased body temperature and fatigue on injured axons due to MS (7, 8).

Here, we review common eye movement disorders in MS and their pathophysiological substrate, how eye movement could be used as model or potentially marker of disease, and what treatment are currently available for ocular motor disorders encountered in MS.

## Eye Movement Disorders in MS

Table 1 summarizes the most common ocular motor manifestations of MS. Demyelinating lesions in the posterior fossa are a frequent cause of ocular motor dysfunction.

**Table 1:** Eye movement disorders of multiple sclerosis.^a^

▪ Strabismus ▫ Exotropia, especially in association with bilateral INO ▫ Esotropia, commonly due to sixth nerve palsy ▫ Vertical deviation, usually a skew deviation in association with INO ▪ Disruption of steady fixation ▫ Gaze-evoked nystagmus ▫ Acquired pendular nystagmus ▫ Upbeat, downbeat, and torsional nystagmus ▫ Positionally induced nystagmus, usually associated with vertigo ▫ Saccadic intrusions and oscillations ▪ Impaired vestibulo-ocular responses, especially vertically associated with INO ▪ Impaired smooth pursuit, optokinetic, and eye-head tracking, especially vertically associated with INO ▪ Disorders of saccades: dysmetria, adduction slowing in INO, ocular flutter ▪ Horizontal gaze paresis or palsy ▪ Dorsal midbrain syndrome

*^a^Adapted with permission from Ref. (6)*.

**Figure 1:**
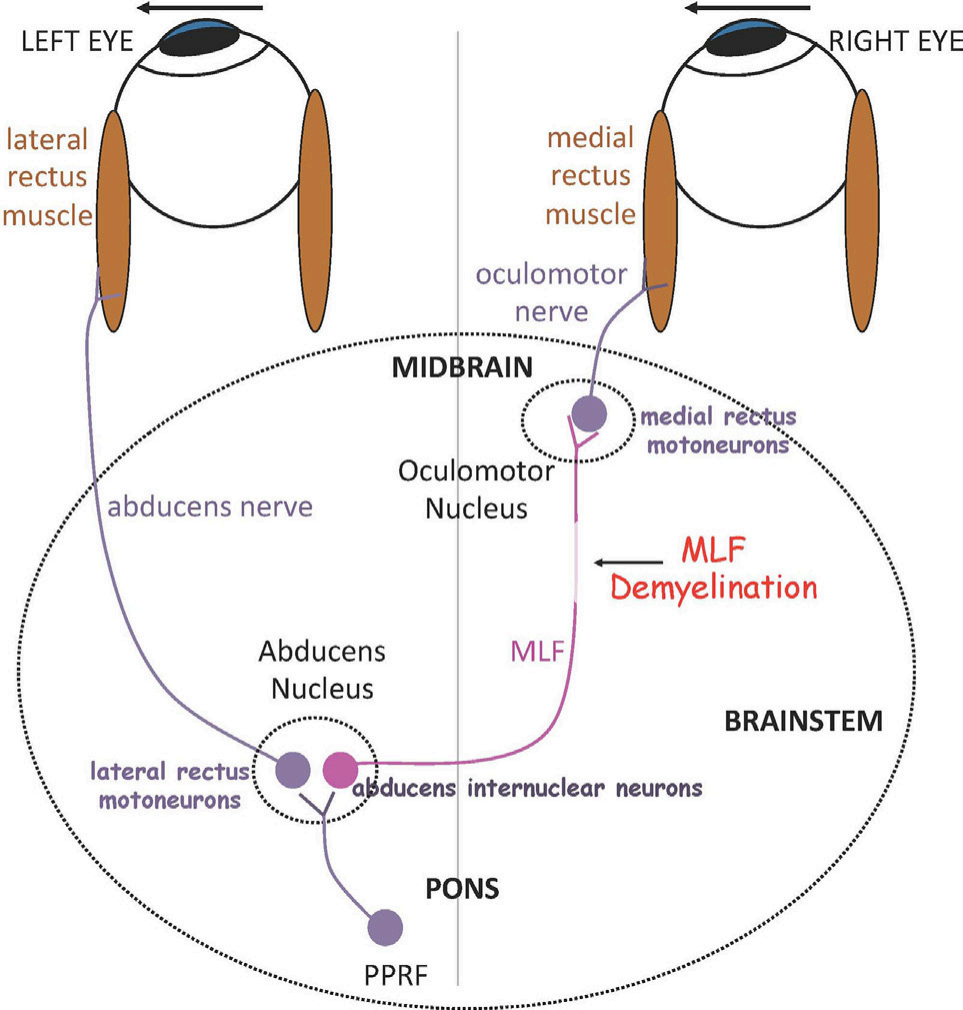
Summary of a simple model for generating horizontal gaze shifts in INO. Premotor excitatory burst neurons lying in the paramedian pontine reticular formation (PPRF), project a pulse of innervation to the abducens nucleus (CN VI). Abducens motoneurons project the pulse of innervation *via* the sixth nerve to the right lateral rectus, which contracts rapidly to generate an abducting saccade of the left eye. Abducens internuclear neurons project the pulse of innervation, *via* the medial longitudinal fasciculus (MLF, internuclear pathway), to medial rectus motoneurons that, in turn, innervate the right medial rectus *via* the third nerve, to generate an adducting saccade of the right eye. If the MLF is demyelinated, signals are low-pass filtered and delayed, affecting the size and timing of the pulse thus causing adducting saccades of the right eye to be slow. *Adapted with permission from Ref. (6).

In the brain stem, demyelination and axonal damage of the medial longitudinal fasciculus (MLF) within the midline tegmentum of the pons (ventral to the fourth ventricle) or the midbrain (ventral to the cerebral aqueduct) results in *internuclear ophthalmoparesis (INO)*, the most common saccadic disorder observed in MS. In INO, binocular coordination (conjugacy) is disrupted with typically slowing of the adducting eye during horizontal saccades (adduction lag), best appreciated during large horizontal saccades and the fast phases of optokinetic reflex testing. On bedside examination, there could be dissociated nystagmus of the abducted eye, which actually consists of saccadic oscillations rather than true nystagmus (9). Patients with INO present with diplopia or more subtle symptoms of blurred vision and visual confusion only during head or head-on-body turns (e.g., during walking or driving) due to a transient break in binocular fusion (10). INO can be associated with skew deviation, a vertical strabismus with hypertropia on the side of the lesion, due to supranuclear disruption of the graviceptive pathways that travel through the MLF and carry utricular and vertical semicircular canals’ input, or the full syndrome of ocular-tilt reaction. The latter is a combination of skew deviation, contralateral head tilt and ocular torsion, and reflects dysfunction of vestibular reactions in the roll plane (11). The role of the MLF in carrying exclusively contralateral posterior semicircular canal signals is confirmed by studies that combine MRI and the video-head-impulse test (12). Patients with unilateral INO may have vertical diplopia due to a non-evident skew deviation, which can be relieved by using a small vertical prism. Skew deviation and OTR can be seen also with lesions independent of the MLF, for example, in the cerebellum or the thalamus. Tilt of the subjective visual vertical, the inner perception of verticality, is very often found in patients with MS (13). In bilateral INO, the vertical vestibulo-ocular reflex and smooth pursuit are usually impaired, as axons in the MLF also carry vestibular and smooth pursuit signals from vestibular nuclei to midbrain nuclei concerned with vertical gaze. Convergence is typically spared in INO, unless the MLF lesion is at a higher level in the midbrain tegmentum. Figure 1 depicts the simple network underlying binocular coordination of horizontal saccades, which is relevant to the pathophysiology of INO (6). To summarize, burst neurons lying in the paramedian pontine reticular formation (PPRF) generate a phasic velocity command called pulse, necessary to initiate the saccade, which is conveyed to two populations of neurons in the abducens nucleus: abducens motor neurons and abducens internuclear neurons. The pulse of innervation travels from abducens motor neurons along axons of the ipsilateral abducens nerve to the lateral rectus muscle, and from the abducens internuclear neurons *via* the MLF to medial rectus motoneurons in the contralateral oculomotor nucleus, which projects to the medial rectus muscle *via* the oculomotor nerve. In normal subjects, the eyes turn rapidly together as an ipsilateral conjugate saccade. Measuring of eye movements allow definition of normal limits for speed, amplitude and latency of abducting and adducting saccades. A physiological adduction lag in the order of ~1–2 ms is generally observed in normal controls (6). Ultimately, INO in MS is due to injury of the MLF, which can no longer conduct high-frequency signals (pulse of innervation) and in turn causes slowing of the adducting eye, which in severe cases can manifest as complete paralysis.

Other brain stem syndromes encountered in MS include fascicular involvement of ocular motor cranial nerves CN III, IV, or VI, the latter sometimes as first clinical event of the disease, and nuclear syndromes such as horizontal gaze palsy, for example, if the PPRF is involved, and one-and-a-half syndrome, a combination of an ipsilateral horizontal gaze palsy with an adduction deficit on the same side. The latter can result from a lesion of the abducens nucleus and the adjacent MLF or, less commonly, from a bilateral INO combined with a unilateral abducens nerve palsy. Dorsal midbrain syndromes can also be encountered with various combinations of upward or downward saccadic gaze palsies, convergence-retraction nystagmus, and convergence impairment.

Patients with MS can present with acute central vestibular syndromes, that might be due to involvement of structures other than the intrapontine eighth nerve fascicle, including the medulla, the cerebellar peduncles, the posterior pontine tegmentum, and the midbrain (14).

The cerebellum and its connections are commonly involved by tissue damage in MS. Saccadic dysmetria is the most common disorder of saccades after INO (13), especially in relation to lesions in the cerebellar peduncles (15). Based on lesion topography, three cerebellar syndromes can be identified. Involvement of *flocculus and paraflocculus*, the so-called vestibulo-cerebellum, usually results into impaired smooth pursuit and inability to suppress the horizontal vestibulo-ocular reflex (VOR) during combined eye-head tracking (16). Gaze-evoked nystagmus (GEN) as well as downbeat nystagmus (DBN) are also found with cerebellar lesions in MS. GEN is a coarse to-and-fro eye oscillation starting with a slow centripetal drift from an eccentric position, followed by a corrective refoveating quick phase. GEN is due to a defect within the neural integrator network, a series of structures that includes the cerebellum, causing inability to hold gaze in eccentric positions (17). DBN, a spontaneous vertical eye oscillation with upward slow phases, is likely due to loss of inhibitory cerebellar control on vertical semicircular canals (18).

Lesions of the *nodulus and uvula* have been shown to cause positional nystagmus, downbeat nystagmus, and periodic alternating nystagmus. Central positional nystagmus, either downbeat or upbeat and clinically presenting as positional vertigo, has been described with demyelinating lesions in the superior cerebellar peduncle (19). Such lesions might cause positional vertigo and nystagmus through disruption of the central otolithic connections between deep cerebellar structures and the vestibular nuclei. Periodic alternating nystagmus (PAN) consists in spontaneous horizontal jerk nystagmus that reverses direction of the quick phases every 2 min. Experimentally, the ablation of the nodulus and ventral uvula in monkeys causes PAN in darkness (20). PAN is well characterized as a disorder of the “velocity storage” process, a sort of “vestibular memory” that physiologically prolongs the otherwise short-lived peripheral vestibular responses. PAN in MS has been linked to demyelination of central vestibular connections at the cerebellar peduncles (21).

Involvement of the *dorsal vermis and fastigial nuclei* typically causes saccadic dysmetria and impaired smooth pursuit, which appears “saccadic” on clinical examination. Dysmetric saccades include hypermetric saccades that overshoot the target, usually due to lesion of the fastigial nuclei, and hypometric saccades that undershoot the target, usually due to lesion of the dorsal vermis. When demyelination affects one fastigial nucleus, lesions are functionally bilateral as axons immediately cross to the contralateral nucleus. Clinically, this is evident as bilateral saccadic hypermetria.

The most common form of nystagmus found in MS is acquired pendular nystagmus (APN), which is cause of significant visual disability. Experimental studies have provided insights into the pathophyisiology of APN in MS, delineating mechanisms that translate into pharmacological treatment. Poor vision and consequent visual input delay along demyelinated visual pathways, for instance due to prior optic neuritis, may not fully explain the occurrence of the high-frequency oscillations that characterize APN. In fact, these oscillations remain unchanged in darkness when visual inputs have no influence on eye movements. Since the oscillations of APN are reset by large saccades, which produce a phase shift of the oscillation (22), it is more likely that APN originates within the neural integrator network in the brain stem and cerebellum (23). The premotor signal responsible for large saccades would basically reset the APN oscillations by silencing some neural integrator neurons that produce the nystagmus. To support this hypothesis, MS patients with APN tend to show more lesions in the paramedian pons in the region of the paramedian tract (PMT) cell groups, part of the neural integrator loop, which would consequently lose normal feedback, becoming unstable and generating the oscillations clinically evident as APN (24).

Multiple sclerosis patients may also suffer from disabling oscillopsia arising from other kind of eye oscillations, such as saccadic intrusions and oscillations, which vary as far as amplitude (e.g., square-wave jerks, macro square-wave jerks, macrosaccadic oscillations). In general, when saccadic intrusions or oscillations show an intersaccadic interval, that is a small pause of usually 200–400 msec between the saccadic to and fro movements, the most likely mechanism is an interruption of the cerebellar feedback on saccades control. On the other hand, saccadic oscillations without intersaccadic interval (e.g., ocular flutter and myoclonus) may derive from an unstable brain stem network and ultimately result from alteration of membrane properties of burst neurons (25–27).

Multiple sclerosis patients may show dysfunction of the higher order control network of eye movements. The integrity of such network can be assessed by means of several experimental paradigms. In the antisaccade task, the most widely used ocular motor test of cognitive control, one is required to inhibit an automatic saccade directed toward a visual stimulus being presented and to generate a saccade of similar amplitude in the opposite direction. A version of this test can be administered manually at bedside (28). The dorsolateral prefrontal cortex (PFC) seems to have a primary role in the inhibition of automatic, reflexive saccades otherwise initiated by the parietal eye fields. MS patients make more mistakes at the antisaccade test and generate saccades with greater latency than controls (29, 30). Such poor performance of MS may correlate also with cerebellar dysfunction as the role of the cerebellum in cognitive control is increasingly recognized (31). MS patients also make mistakes when required to generate saccades toward a remembered target, the so-called memory guided saccades, which is thought to reflect a deficit of working memory. During this task, their saccades are also inaccurate, especially when asked to execute a memorized sequence of target jumps (32). Finally, saccades made in response to predictable target jumps are usually hypometric in MS, and latencies are increased for saccades toward visual targets presented at random locations along with a visual distracter (33). These abnormalities also reflect inability to maintain inhibitory control through the PFC and its connections to thalamocorticostriatal circuits (34).

## Eye Movements as Model and Marker of Disease in MS

Measurement and modeling of eye movements in MS has proven helpful in documenting a vast range of abnormalities and shed light onto specific pathogenic mechanisms, often providing the rationale for testing pharmacological intervention.

Internuclear ophthalmoparesis is perhaps the most useful application of eye movement measurement and modeling. INO in MS has been studied and quantified in several ways. A traditional approach, using infrared, search coil, or videooculography based techniques (6), is to compare the peak velocity, or peak acceleration, of the abducting eye versus the adducting eye during saccades. The abduction/adduction ratios of peak velocity or peak acceleration is consistently increased in patients with INO compared to normal controls (35–37). Studies have shown that mild INO may go undetected on bedside examination, which of course could have clinical implications when trying to establish a diagnosis or quantify disability (38). Another method to quantify INO is to calculate the abducting/adducting eye amplitude ratio at the time the normal abducting eye first reaches the target (first pass amplitude), and not at the end of the saccade, when a mildly affected adducting eye may eventually lands on target (39). A third method consists in plotting velocity values as a function of changes of eye position. This phase plane approach is particularly useful when attempting to distinguish true INO, for example, due to MS, from its mimickers such as a pseudo-INO due to myasthenia gravis (40). The phase plane plots show that in true INO the abducting and adducting eyes are dysconjugate from the onset of the saccade, while in pseudo-INO the initial portion of the saccade shows normal binocular coordination, only later in the movement followed by obvious dysconjugacy.

The significance of modeling INO in MS resides in the fact that the MLF is an accessible discrete pathway that lends itself as a microcosm of MS pathology, in particular demyelination and axonal loss ultimately responsible for conduction delay. INO as a reductionist model for decreased fidelity of neural conduction, has been used to study the effect of body temperature changes (Uhthoff’s phenomenon) and of motor fatigue in MS. Thus, with increase in core body temperature, horizontal binocular conjugacy worsens in patients with INO (7), as it does when they are asked to make horizontal saccades continuously over several minutes (a fatigue test) (8). The approach of using eye movements to better characterize and follow over time disabling symptoms like fatigue in MS, is supported by studies showing changes of both exogenous and endogenous saccadic peak velocity, latency and amplitude in patients who report symptoms of fatigue (41, 42). The effects of ocular motor fatigue on INO can be characterized not only in terms of decreased amplitude of the saccadic pulse signal for the adducting eye but also in terms of its delayed delivery through the injured MLF (43). Both these behaviors can be reproduced by manipulating conduction gains and delays at the MLF site, within a mathematical model of the faulty circuitry responsible for INO (43).

Can INO be used as a biomarker of axonal and myelin integrity in MS? Modern applications of high-resolution MRI such as diffusion tensor imaging (DTI) techniques are able to capture architectural changes of discrete white matter tracts, including the MLF, due to myelin and axonal pathology. Coupling DTI-based neuroimaging with eye movement measurement, for example, with video oculography, may help characterize axonal integrity and myelin status and quantify tissue injury in the MLF (44–47). With a similar approach, latency of onset of vestibulo-ocular reflex (eVOR) recorded using search-coil, was studied in relation to lesion length and DTI metrics of MLF, and was found to be more prolonged with greater extent of MLF lesions (48). This study also provided direct measurement of axonal conduction velocity within lesions involving the MLF, which was reduced below levels predicted for natively myelinated and remyelinated axons. Such approach of studying the MLF as a composite structural-functional biomarker of axonal and myelin status could be used to assess response to therapies aimed at enhancing recovery and fidelity of axonal transmission in MS. In this regard, preliminary data in three INO patients treated with the potassium channel blocker dalfampridine showed changes in horizontal saccadic conjugacy, consistent with improved transmission of the neural pulse responsible for adducting movements (49).

Can eye movements be used as a biomarker for cognitive compromise in MS? As discussed above, eye movements can be affected by dysfunction of higher-order networks that control cognition. Such abnormalities, evident mostly as errors and increased saccadic latencies at the antisaccade and memory-guided tests, may be detected in the early stages of the disease and their deterioration over time correlates with neuropsychological test scores (34). Thus, eye movements could represent a useful tool to interrogate integrity of cortical and subcortical networks, and possibly their cerebellar connections, that are involved with attention, working memory and executive function.

In summary, while Inflammatory, demyelinating, and neurodegenerative pathology in multiple sclerosis affect both afferent and efferent visual function, the afferent visual system has been utilized to a significant larger extent as a model system for MS. A simple clinical tool such as low-contrast letter acuity testing has been shown to clearly capture visual dysfunction and visual disability in MS, and to correlate with structural changes on optical coherence tomography (OCT) and disease burden on MRI (50). The introduction of OCT has been a breakthrough in the field of MS: changes in the thickness of peripapillary retinal nerve fiber layer are felt to represent axonal damage, whereas loss of macular volume and thinning of retinal ganglion cell layer are viewed as evidence of neuronal pathology. The afferent visual system has the advantage of being probably more accessible for interrogating status and severity of disease and testing of possible agents for neuroprotection and repair in MS (51). While eye movements assessment and quantification may require special equipment and a particular clinical expertise, several studies have shown that, for instance, certain features of saccades in INO and in cognitive function, have the potential to be validated as markers of disease and treatment outcome measures.

## Treatment of Eye Movement Dysfunction in MS

Several pharmacological agents have been employed to treat acquired eye movement disorders, for example, pendular or downbeat nystagmus. When these disorders are secondary to multiple sclerosis, localization of causative lesions by MRI can help elucidate the pathophysiological mechanisms responsible for the eye movement impairment and drive therapeutic choices. Acquired pendular nystagmus (APN) is a classic example. As discussed above, APN is likely due to an unstable neural integrator loop, which includes the region of the paramedian tracts, where a higher disease load can be found in MS patients. Drugs that depolarize the NI cells, improving membrane stability, may reduce APN amplitude (52). Gabapentin (1,200 mg/day) and memantine (15–60 mg/day), blockers of alpha-2-delta calcium channels and glutamate receptors, respectively, reduce GABAergic inhibition of cerebellar Purkinje cells causing indirect depolarization of the cells of a key NI structure, the nucleus prepositus hypoglossi (53, 54). Downbeat nystagmus (DBN) in MS, usually caused by lesions of the cerebellar flocculus, has been shown to respond to oral clonazepam (0.5 mg 3 times daily), baclofen (10 mg 3 times daily), and gabapentin. Randomized controlled trials have shown that the potassium channel blockers 3,4-diaminopyridine (3,4-DAP) had a significant effect of peak slow-phase velocity (55). 4-aminopyridine (4-AP), which restores the function of the vertical and horizontal neural integrator, should be preferred to 3,4-DAP because it crosses the blood–brain barrier more easily. Because of short half-life, a sustained release form of 4-AP such as dalfampridine (10–20 mg/day) is recommended (56–58). 4-AP has been shown effective also in upbeat nystagmus (UBN) and central positional nystagmus (59, 60), while baclofen (5–10 mg 3 times daily) can also be considered for UBN. Periodic alternating nystagmus (PAN), that could arise in MS from demyelination of central vestibular connections at the cerebellar peduncles, has been treated with oral baclofen (5–10 mg 3 times daily) in case reports. The effects of baclofen on the vestibular nuclei or vestibulocerebellum likely depend on GABA mechanisms.

**Figure 2:**
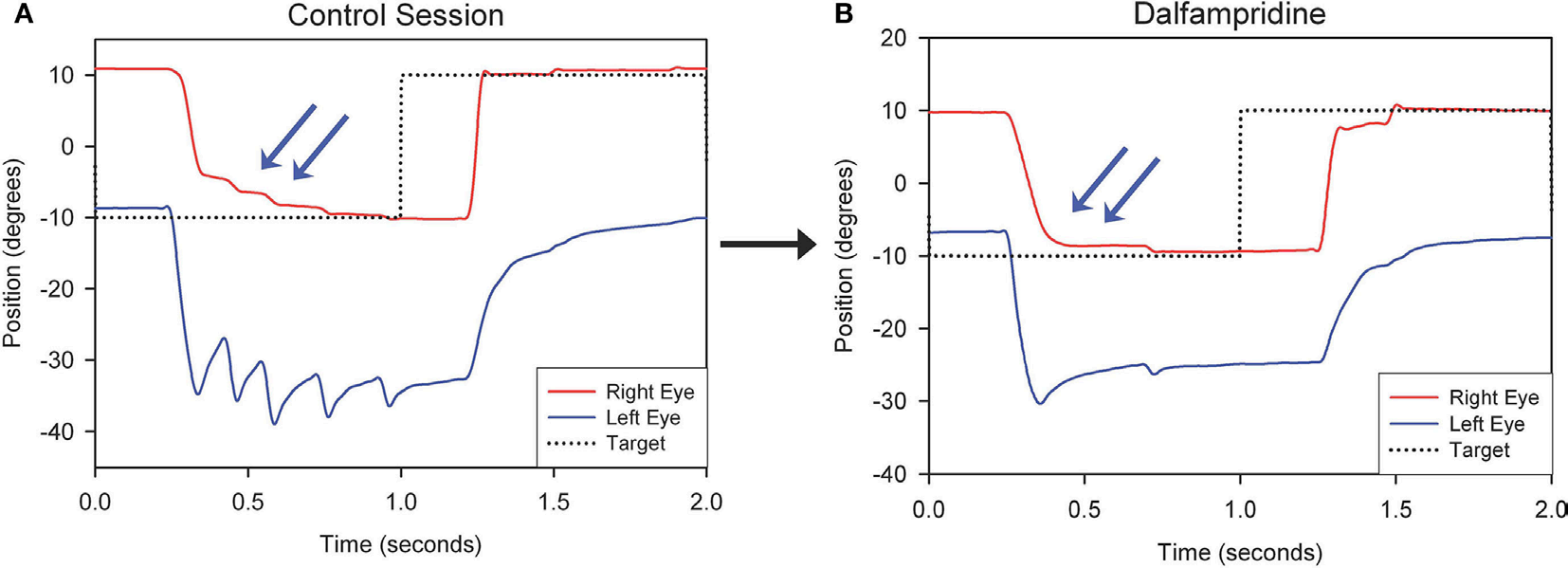
Representative horizontal leftward saccade before **(A)** and after Dalfampridine **(B)** in one patient. **(A)** Particularly for the leftward movement the adducting eye requires several small saccades to acquire the target (arrows) and the abducting eye shows dissociated nystagmus. **(B)** The right eye requires less adducting saccades to acquire the target (arrows) and dissociated nystagmus intensity is decreased. Positive values indicate rightward movements, negative values indicate leftward movements. *Adapted with permission from Ref. (49).

Therapeutic strategies for eye movement disorders in MS may not be limited to treatment of nystagmus. Thus, as mentioned above, a study of three MS patients with chronic internuclear ophtalmoplegia (INO) showed that dalfampridine improved horizontal saccadic conjugacy, as recorded with video oculography, with one of the patients reporting actual clinical improvement of quality of vision (Figure 2). A double-blind placebo-controlled crossover trial of dalfampridine to treat INO and associated ocular motor fatigue due to MS is ongoing (ClinicalTrials.gov NCT02391961). Finally, non-pharmacological interventions, such as the use of base-down prisms for DBN, which is usually less intense in upward gaze, or prisms to compensate for skew deviation causing vertical diplopia in INO, should always be considered.

## Conclusion

We have reviewed the most common eye movement disorders in MS and discussed known pathophysiological correlate for each of them. Study of eye movements in MS is particularly valuable as use of conventional and upcoming non-conventional imaging techniques coupled with eye movement recording can provide insights into mechanisms and status of disease. Eye movements abnormalities in MS are once again a promising tool with the tangible potential of serving as biomarkers of early disease, monitoring tools, and outcome measures for testing new treatments, including remyelinating agents.

## Author Contributions

AS: wrote draft and final version of the article, adapted, and completed table and figures. CC: and MM: critical reading, draft editing, and helped references.

## Disclaimer

The contents do not represent the views of the U.S. Department of Veterans Affairs or the United States Government.

## Conflict of Interest Statement

The authors declare that the research was conducted in the absence of any commercial or financial relationships that could be construed as a potential conflict of interest.
